# Associations of Children’s Appetitive Traits with Weight and Dietary Behaviours in the Context of General Parenting

**DOI:** 10.1371/journal.pone.0050642

**Published:** 2012-12-05

**Authors:** Gerda Rodenburg, Stef P. J. Kremers, Anke Oenema, Dike van de Mheen

**Affiliations:** 1 IVO Addiction Research Institute, Rotterdam, The Netherlands; 2 Erasmus Medical Center, Rotterdam, The Netherlands; 3 Department of Health Promotion, Maastricht University, Maastricht, The Netherlands; University of Minnesota, United States of America

## Abstract

**Background:**

Individual variations in child weight can be explained by genetic and behavioural susceptibility to obesity. Behavioural susceptibility can be expressed in appetite-related traits, e.g. food responsiveness. Research into such behavioural factors is important, as it can provide starting points for (preventive) interventions.

**Objectives:**

To examine associations of children’s appetitive traits with weight and with fruit, snack and sugar-sweetened beverage intake, and to examine whether parenting style interacts with appetite in determining child weight/intake.

**Methods:**

Data were used from 1275 children participating in the INPACT study in 2009–2010, with a mean age of 9 years in 2009. Their height and weight were measured to calculate body mass index (BMI). Parents completed a questionnaire to measure children’s appetitive traits, children’s dietary intake and parenting style. Child BMI z-scores, fruit, snack and sugar-sweetened beverage intake were regressed on appetitive traits. Moderation by parenting style was tested by adding interaction terms to the regression analyses.

**Results:**

Food-approaching appetitive traits were positively, and food-avoidant appetitive traits were negatively related to child BMI z-scores and to child fruit intake. There were no or less consistent associations for snack and sugar-sweetened beverage intake. Authoritative parenting voided the negative association between food fussiness and fruit intake, while neglecting parenting strengthened the positive association between food-approaching appetitive traits and weight.

**Conclusions:**

Early assessment of appetitive traits could be used to identify children at risk for overweight. As parenting style can moderate the associations between appetitive traits and weight/intake in a favourable way, parents are a promising target group for preventive interventions aimed at influencing the effect of appetitive traits on children.

## Introduction

The prevalence of overweight and obesity among children has increased rapidly over the last decades [1]. On the population level, part of the explanation can be found in the obesogenic environment [2], which is characterized by constant availability of cheap energy-dense food and advancement of sedentary lifestyles. However, the changed environment cannot explain individual variations in body weight in children, which exist and will persist. There is evidence for genetic susceptibility [2], [3] and behavioural susceptibility to obesity, the latter reflected in appetite-related traits [4]. Experimental studies using behavioural tests, as well as large-scale observational studies using questionnaires, show that food-approaching appetitive traits (e.g. food responsiveness) are positively associated with child overweight, while food-avoidant appetitive traits (e.g. food fussiness) are negatively associated with child overweight [5]–[7]. Observational studies have shown that these associations were graded: individual variations in appetite were related to body weight in general and not exclusively to overweight or obesity [8]–[15]. This implies that early assessment of appetitive traits could identify ‘food approaching’ children, who have a higher risk of becoming obese and for whom prevention interventions could be developed to modify their eating style.

Observational studies on children’s appetitive behaviours have used various instruments, including the Dutch Eating Behaviour Questionnaire (DEBQ) [10], the Children’s Eating Behaviour Inventory (CEBI) [16] and the Child Eating Behaviour Questionnaire (CEBQ) [17]. These studies almost exclusively examined associations with child (over)weight [4]–[6], [9], [11]–[13], [16], [18]–[20] and seldom with child dietary behaviours [21]–[24]. However, to understand the mechanisms by which appetitive traits affect child weight it is important to include them.

Some studies on children’s appetitive behaviours incorporated parental feeding practices [18], [25], [26]. Insight into such parental influences on appetite is promising for intervention development targeted at parents, as parents play a key role in shaping the food home environment [27], [28], e.g. by creating availability of and accessibility to foods, by setting norms and values, and by their own behaviour (modelling). However, parents also influence their child’s behaviour in a more general way by expressing a certain parenting style, which generates the environmental and emotional context for child rearing [29]. A recent review showed that children raised in authoritative homes, characterized by high involvement and high control, ate more healthy and had lower body mass index (BMI) levels than children raised in authoritarian, permissive or neglectful homes [30]. The review also mentioned findings from moderation studies, indicating that parenting style has a differential impact on children’s weight-related outcomes, depending on (for example) child characteristics. This is in line with the ecological systems theory [31] and implies that the impact of children’s appetitive traits on dietary intake and weight may differ depending on the parents’ parenting style. Because authoritative parenting is seen as a protective factor for unhealthy eating and overweight, it may also affect the relationship between children’s appetitive trait and weight/intake in a favourable way, e.g. by attenuating or voiding the positive relationship between a food-approaching appetite and weight.

Studies on heritability of appetitive behaviours support a strong genetic component [32], and appetite can be seen as a stable personality trait [33]. This suggests that appetitive traits influence child intake/weight, rather than that they are consequences of a child’s intake/weight. This observation is supported by a limited number of longitudinal studies in which baby’s appetitive traits were prospectively related to weight gain (see e.g. [34]). To our knowledge, no prospective studies have incorporated child intake to explore whether the strength of associations changes over time, and whether child appetite traits predict changes in child intake.

The present study examines cross-sectional and longitudinal (one-year follow-up) associations of children’s appetitive traits with weight and with dietary behaviours in a large, community-based sample of children aged 8–11 years. We chose to include obesity-reducing, i.e. child fruit intake, as well as obesity-inducing dietary behaviours, i.e. child snack and sugar-sweetened beverage (SSB) intake [35]. We also examined whether the potential associations between children’s appetite and weight/intake are moderated by parenting style. It was hypothesized that 1) food-approaching traits would positively relate to child fruit intake, snack intake, SSB intake and weight, while food-avoidant traits would negatively relate to these measures, and that 2) the potential associations would be moderated by authoritative parenting in a favourable way; e.g. authoritative parenting would attenuate or void the potential positive association between food responsiveness and child snacking, and the potential negative association between food fussiness and fruit intake.

## Methods

### Study Design, Participants and Procedure, Including Ethics Statement

Data for this study were retrieved from the longitudinal IVO Nutrition and Physical Activity Child cohorT (INPACT), for which approval was obtained from the Ethical Committee of the Erasmus MC (University Medical Center Rotterdam). INPACT is an observational study (initiated in 2008) focusing on modifiable determinants of overweight in the home environment of children in the Netherlands aged 8–12 years. The study included four assessments, in which qualified research assistants measured the children’s height and weight at school, and primary caregivers completed a questionnaire at home. Questionnaires recorded data on dietary intake of the child, child appetitive behaviours, and potentially relevant home environmental factors, including the primary caregiver’s dietary intake, parenting style and socio-demographic variables. Assessments took place with a one-year time interval, and started in the autumn of 2008 (baseline).

INPACT was conducted among primary school children in southern Netherlands (Eindhoven area). In recruiting the schools in 2008, we collaborated with the Municipal Health Authority for Eindhoven and surrounding area (GGD Brabant-Zuidoost). The Municipal Health Authority invited all general primary schools in their service area to participate in the INPACT study. Of the 265 schools invited, 91 took part. The response rate from rural and urban schools was equal. The primary caregivers of third-grade students (aged ±8 years) were invited to participate in the cohort study, together with their child. Of the 2948 parent-child dyads invited, 1839 (62.4%) gave written informed consent to participate in the INPACT study for four years.

The present study was based on data from 2008 (baseline), 2009 (second assessment) and 2010 (third assessment). Socio-demographic variables and general parenting style were measured at baseline. The child’s appetitive behaviour was measured in 2009, while child fruit intake, snack intake, SSB intake and weight were measured in 2009 and 2010. Parent-child dyads who completed the parent questionnaires from baseline to 2010, and had valid child height and weight data in 2009 and 2010 were included in the present study, resulting in 1275 parent-child dyads (69% of the original cohort). Logistic regression analyses on selective dropout from baseline to 2010 showed that parent-child dyads who were not native Dutch dropped out more often. There was no selective dropout regarding child age/gender and parental education level.

### Sample Characteristics

At baseline (n = 1839), 7% of the children were underweight, 79% had a normal weight and 14% were overweight, of which 3% obese. The prevalence of overweight and obesity was similar to Dutch prevalence rates among primary school children [36]. The age of the children was 8 (77%) or 9 (20%) years (range 7–10, mean = 8.2, SD = 0.5 years). Boys (50.5%) and girls (49.5%) were represented in almost equal numbers. Of all children, 17% were from a non-Dutch ethnic background with one or both parents born abroad, of which 9% from non-western countries and 8% from western countries. Of all primary caregivers, 21% had finished education at a low level, 45% at a medium level, 32% at a high level, and 2% at a non-specified level (see Measures section for classification system used). Of the primary caregivers 1% was underweight, 66% had a normal weight and 33% were overweight, of which 9% were obese.

### Measures

#### Children’s appetitive behaviour

Appetitive behaviour was measured using a validated Dutch translation [12] of the Children’s Eating Behaviour Questionnaire (CEBQ), designed by Wardle et al. [17]. This 35-item measure assessed eight appetitive traits: food responsiveness (FR), enjoyment of food (EF), emotional overeating (EOE) and desire to drink (DD) as ‘food-approaching’ appetitive traits, and satiety responsiveness (SR), slowness in eating (SE), emotional undereating (EUE) and food fussiness (FF) as ‘food-avoidant’ appetitive traits. The original measure, as well as the Dutch translation, proved to possess adequate to good internal consistency [12], [17]. The CEBQ is generally regarded as the most comprehensive instrument to assess children’s eating styles, and correlates well with behavioural tests designed to measure such appetitive traits [6].

Missing data on the CEBQ items (1.6% at the highest) were imputed using the mean value of respondents without a missing value. Table 1 presents additional information on number of items, example items, response options, Cronbach’s alphas, and means and standard deviations (SDs) of the appetitive behaviours.

**Table 1:** Descriptives and scale information of child eating behaviours and parenting style dimensions.

Category	Concept	Measurement year (n)	# items: example item	Answering scale^i^	Cronbach’s α^ii^	Mean (SD)	Range of scores
*Child Eating* *Behaviours*	Food Responsiveness	2009 (1547)	5: ‘Given the choice, my child would eat most ofthe time.’	A	0.79	1.9 (0.7)	1.0 to 5.0
	Enjoyment of Food	2009 (1547)	4: ‘My child enjoys eating.’	A	0.79	3.4 (0.7)	1.0 to 5.0
	Emotional Overeating	2009 (1547)	4: ‘My child eats more when anxious.’	A	0.75	1.6 (0.6)	1.0 to 4.8
	Desire to Drink	2009 (1547)	3: ‘My child is always asking for a drink.’	A	0.83	2.0 (0.7)	1.0 to 5.0
	Satiety Responsiveness	2009 (1547)	5: ‘My child gets full before his/her meal is finished.’	A	0.73	2.6 (0.6)	1.0 to 4.8
	Slowness in Eating	2009 (1547)	4: ‘My child eats slowly.’	A	0.80	2.5 (0.8)	1.0 to 5.0
	Emotional Undereating	2009 (1547)	4: ‘My child eats less when s/he is upset.’	A	0.78	2.3 (0.8)	1.0 to 5.0
	Food Fussiness	2009 (1547)	6: ‘My child decides that s/he does not like food,even without tasting it.’	A	0.89	2.8 (0.9)	1.0 to 5.0
						**Sum score (SD)**	
*Parenting style dimensions*	Support	2008 (1839)	7: ‘When my child gets a low grade in school,I offer to help him/her’	B	0.71	11.0 (2.4)	1.7 to 14.0
	Behavioural control	2008 (1839)	7: ‘I try to know where my child goes after school’	B	0.72	9.5 (4.2)	−5.0 to 14.0
	Psychological control	2008 (1839)	8: ‘I make my child feel guilty when he/she getsa low grade in school’	B	0.72	−6.7 (4.1)	−16.0 to 16.0

a Answering scale A: never (1) to always (5); answering scale B: completely disagree (−2) to completely agree (+2).

b The reliability of the child eating behaviour scales was assessed by calculating Cronbach’s alphas (internal consistency) and (average) corrected item-total correlations, which indicate the degree to which an individual item relates to the total scale score. Corrected item-total correlations above 0.30 are regarded as good and below 0.15 as unreliable. Average corrected item-total correlations in our study were good and ranged from 0.56 to 0.71. None of the corrected item-total correlations was below 0.3 (lowest value was 0.37 for a Satiety Responsiveness-item).

#### Children’s intake

Child fruit, snack and SSB intake were measured with a questionnaire that was based on validated Food Frequency Questionnaires [37], [38]. The primary caregivers reported how many days in a normal week their children consumed 1) fruit (fresh, bottled and/or canned; no juice), 2) savoury snacks (e.g. potato crisps, peanuts and sausage rolls) in between meals, 3) sweet snacks (e.g. candies, chocolates and candy bars) in between meals, 4) cake or large biscuits in between meals, and 5) SSBs. Answering categories ranged from ‘none or less than 1 day a week’ to ‘7 days a week’. Additionally, they reported the number of servings consumed by their children on such a day. For fruit, answering categories ranged from ‘0 pieces per day’ to ‘more than 3 pieces per day’, by increments of half a piece of fruit. Reported consumption of more than 3 pieces per day (n = 12) was recoded as 4 pieces. For savoury snacks, sweet snacks and cake or large biscuits, answering categories ranged from 0 to 10 servings a day. For SSBs, answering categories ranged from ‘0 glasses per day’ to ‘more than 5 glasses per day’, by increments of half a glass. It was specified that one glass equals 200 ml; one can equals 330 ml or 1.5 glasses; one bottle equals 500 ml or 2.5 glasses. Reported consumption of more than 5 glasses per week (n = 7) was recoded as 6 glasses. Total child fruit and SSB intake were expressed in servings per week and calculated by multiplying frequency and quantity. Total child snack intake was also expressed in servings per week and calculated by multiplying frequencies of savoury snacks, sweet snacks and cakes with their corresponding quantities, and summing these scores. Missing values on child fruit, snack and SSB intake were not imputed, because of the low number of missing values (1.0% at the highest, for child snacking).

#### Children’s weight

Child BMI was based on the child’s height and weight: i.e. weight (kg)/height (m)^2^, as measured by the qualified research assistants. Children were measured at school according to standard procedures in light clothing without shoes, to the nearest 0.1 kg and 0.1 cm. BMI z-scores were calculated [39] based on age and gender-specific values from the 1997 National Growth Study in the Netherlands [40].

#### Parenting style

Parenting style was measured using the Dutch translation [41] of an instrument based on earlier work by Steinberg et al. [42], [43], which is used in many studies worldwide [41], [44]–[46]. This 22-item measure assessed three parenting-style dimensions: support, behavioural control and psychological control (see Table 1 for details). Based on these dimensions, we constructed five parenting styles by dichotomising the sample on each dimension (median-split) and by examining the three dimensions simultaneously [47], [48]: the authoritative (high support, high behavioural control, low psychological control), permissive (high support, low behavioural control, low psychological control), authoritarian (low support, high behavioural control, low psychological control), rejecting (low support, low behavioural control, high psychological control) and neglecting (low support, low behavioural control, low psychological control) parenting style.

#### Confounders

Measured confounders included child’s gender, age and ethnic background, parental education level, parental fruit, snack and SSB intake, and parental BMI. To assess the child’s ethnic background, the primary caregiver reported the country of origin of both parents. According to standard procedures of Statistics Netherlands [49], a child was classified as native Dutch if both parents were born in the Netherlands, as a western immigrant if at least one parent was born outside the Netherlands but inside Europe (including former Yugoslavia and the Soviet Union), North America, Oceania, Indonesia or Japan, and as a non-western immigrant if at least one parent was born in Turkey, Africa, Latin America or Asia. The primary caregiver also reported his/her highest level of education. According to international classification systems, parental education level was defined as low (primary school and lower vocational/lower general secondary education), medium (intermediate vocational education, higher general secondary education and university preparatory), high (higher vocational education and university), or non-defined.

Parental fruit, snack and SSB intake were measured and calculated in the same way as child fruit, snack and SSB intake. To assess parental BMI, the primary caregiver reported his/her own height and weight, and that of his/her partner. He/she also reported whether he/she and the partner were the child’s biological parents. Maternal and paternal BMI (for biological parents only) were calculated on the basis of their answers (n_maternal BMI_ = 1204, 5.6% missing; n_paternal BMI_ = 1058, 17.0% missing). To maintain statistical power, missing values on maternal and paternal BMI were imputed using the group mean.

### Strategy for Analyses

To describe the study population, we computed means, SDs and/or proportions for the socio-demographic variables, CEBQ scales, parenting style dimensions, child dietary behaviours and child BMI z-scores.

Separate linear regression analyses were performed to establish the longitudinal relationship between CEBQ scales and child intake/child BMI z-scores in 2010, adjusted for child age, gender, ethnic background and parental education level. In models with child intake as dependent variable (e.g. child fruit consumption), we also controlled for child BMI in 2009 and parental intake in 2010 (i.c. parental fruit consumption). In models with child BMI z-scores as dependent variable, we controlled for the socio-demographic variables and parental BMI in 2010. In these models, underweight children in 2009 (91 of 1275 children) were excluded to prevent distortion of the results (for underweight children, an increase in BMI would be favourable, while it would be unfavourable for normal, overweight and obese children). International cut-off scores were used to determine whether a child was underweight [39].

To determine whether CEBQ scales predicted changes in child intake and BMI z-scores between 2009 and 2010, we repeated the linear regression analyses, additionally adjusted for child intake in 2009 and child BMI z-scores in 2009, respectively. Finally, to explore whether the longitudinal associations between CEBQ scales in 2009 and child intake/weight in 2010 were similar to cross-sectional associations, we also performed cross-sectional linear regression analyses (CEBQ scales and child intake/weight in 2009), applying the same adjustment procedure as in the longitudinal analyses.

In the final set of regression analyses we examined whether parenting style moderated significant longitudinal associations between CEBQ scales and (changes in) child intake/child weight. Moderation was tested by adding interaction terms to the regression analyses. If interaction terms were significant (p<0.05), stratified analyses were conducted.

All analyses were conducted using SPSS version 18.0.

## Results

CEBQ and parenting style dimensions are described in Table 1. Children had an average weekly fruit consumption of 7.3 (SD = 4.2) pieces in 2009 and 6.9 (SD = 4.3) pieces in 2010, an average weekly snack intake of 9.8 (SD = 5.8) pieces in 2009 and 9.9 (SD = 6.1) pieces in 2010, an average weekly SSB intake of 9.2 (SD = 8.2) glasses in 2009 and 8.9 (SD = 8.2) glasses in 2010, and an average BMI z-score of 0.2 (SD = 0.9) in both 2009 and 2010 when underweight children were excluded.

Results of the regression analyses with child intake/child BMI z-scores in 2010 as dependent variable (Table 2, column ‘β_2010_’) showed that all food-approaching subscales were positively associated with child BMI z-scores. The food-approaching subscales FR and EF were positively associated with child fruit intake, but EF was negatively associated with child snack intake. DD was positively associated with child snack intake. All food-avoidant subscales were negatively associated with child BMI z-scores and child fruit intake, but SR was positively associated with child snacking and SE positively associated with child SSB intake. Results of the regression analyses with child intake/child BMI z-scores in 2009 as dependent variable (Table 2, column ‘β_2009_’) were generally similar to those for 2010.

**Table 2:** Associations (standardized regression coefficient) of child eating behaviours (2009) with child fruit intake, snack intake, SSB intake and BMI z-scores in 2009, in 2010 and in 2010, controlled for 2009 value.^1^

	Child fruit intake^2^			Child snacking^3^			Child SSB intake^4^			Child BMI z-scores^5^		
	β_2009_ 6	β_2010_ 7	β_2010–__2009_ 8	β_2009_^f^	β_2010_^g^	β_2010-__2009_^h^	β_2009_^f^	β_2010_^g^	β_2010–__2009_^h^	β_2009_ 9	β_2010_ 10	β_2010–__2009_ 11
Food responsiveness (FR)	0.06*	0.06*	0.02	0.05	0.03	0.03	0.03	0.00	−0.01	0.33***	0.31***	0.00
Enjoyment of food (EF)	0.13***	0.15***	0.06**	−0.06*	−0.06*	−0.02	−0.01	−0.02	−0.02	0.17***	0.17***	0.01
Emotional overeating (EOE)	−0.01	−0.03	−0.03	0.07**	0.03	0.01	0.01	−0.02	−0.01	0.18***	0.18***	0.01
Desire to drink (DD)	−0.08**	−0.05	0.01	0.09***	0.07*	0.02	0.07**	0.04	0.03	0.11***	0.10***	0.00
Satiety responsiveness (SR)	−0.17***	−0.12***	0.00	0.09***	0.06*	0.00	0.05	0.04	0.03	−0.17***	−0.16***	0.01
Slowness in eating (SE)	−0.10***	−0.08**	−0.01	0.05	0.02	−0.02	0.02	0.07**	0.07**	−0.15***	−0.13***	0.02
Emotional undereating (EUE)	−0.07**	−0.07**	−0.02	0.01	−0.01	−0.01	0.01	−0.01	0.00	−0.09**	−0.09**	−0.01
Food fussiness (FF)	−0.16***	−0.14***	−0.02	0.05	0.05	0.02	0.03	0.04	0.03	−0.08**	−0.08**	0.00

a *correlation is significant at the 0.05 level (2-tailed);

** correlation is significant at the 0.01 level (2-tailed);

*** correlation is significant at the 0.001 level (2-tailed).

2 n = 1248 for 2009, n = 1245 for 2010 and n = 1244 for 2010-2009; n deviates from sample size in table 2 because of missing values on control variables.

3 n = 1230 for 2009, n = 1233 for 2010 and n = 1217 for 2010-2009; n deviates from sample size in table 2 because of missing values on control variables.

4 n = 1248 for 2009, n = 1239 for 2010 and n = 1238 for 2010-2009; n deviates from sample size in table 2 because of missing values on control variables.

5 n = 1163 for 2009, 2010 and 2010-2009; n deviates from sample size in table 2 because of missing values on control variables; underweight children in 2009 were excluded from analyses with child BMI z-scores as dependent variable. Repeated analyses including underweight children resulted in similar findings.

6 models adjusted for age, gender, SES, ethnicity, child BMI and parental fruit/snack/SSB intake in 2009; β = standardized regression coefficient.

7 models adjusted for age, gender, SES, ethnicity, child BMI in 2009 and parental fruit/snack/SSB intake in 2010.

8 models adjusted for age, gender, SES, ethnicity, child BMI in 2009, parental fruit/snack/SSB intake in 2009 and 2010, and child fruit/snack/SSB intake in 2009.

9 models adjusted for age, gender, SES, ethnicity and parental BMI in 2009.

10 models adjusted for age, gender, SES, ethnicity and parental BMI in 2010.

11 models adjusted for age, gender, SES, ethnicity, parental BMI in 2009 and 2010, and child BMI z-scores in 2009.

Results of regression analyses with child intake/child BMI z-scores in 2010 as dependent variable in which we additionally adjusted for child intake/child BMI z-scores in 2009 (Table 2, column ‘β_2010-2009_’), showed that EF predicted a small increase in child fruit consumption between 2009 and 2010, and that SE predicted a small increase in child SSB intake between 2009 and 2010.

**Figure 1:**
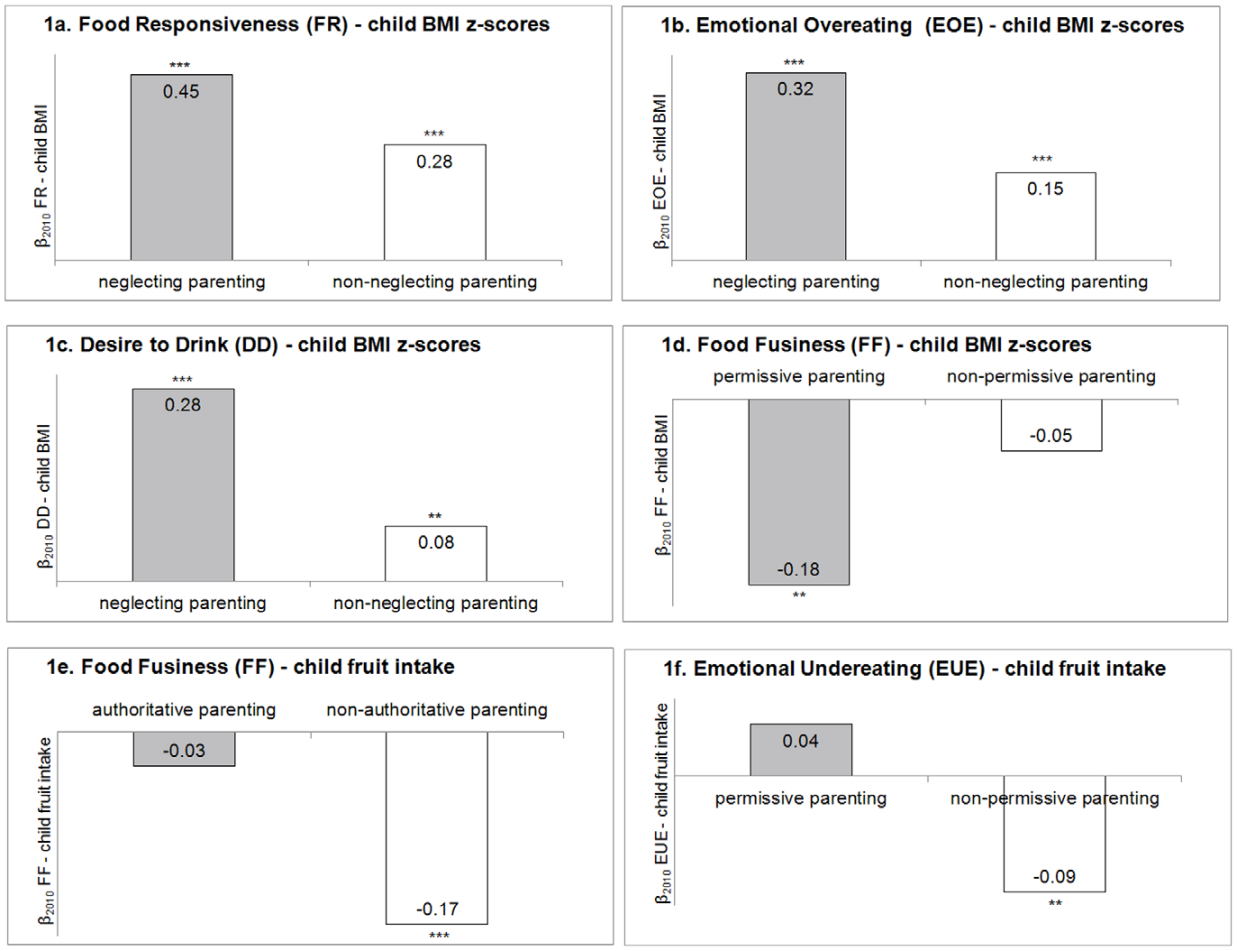
Significant moderating effects of parenting styles on the longitudinal associations between CEBQ subscales and child intake/child BMI z-scores in 2010. Moderation testing was performed on significant longitudinal associations between CEBQ scales and (changes in) child intake/child weight (Table 2, column ‘β2010’ and column ‘β2010-2009’). p_interaction term Figure 1a_ = 0.023; p_interaction term Figure 1b_ = 0.082; p_interaction term Figure 1c_ = 0.018; p_interaction term Figure 1d_ = 0.068; p_interaction term Figure 1e_ = 0.020; p_interaction term Figure 1f_ = 0.038. * correlation is significant at the 0.05 level (2-tailed); ** correlation is significant at the 0.01 level (2-tailed); *** correlation is significant at the 0.001 level (2-tailed).

Moderation analyses and subsequent stratified analyses revealed that the negative associations of FR, EOE and DD with child BMI z-scores one year later were strengthened when parents had a neglecting parenting style (see Figure 1a–c). The negative association between FF and child BMI z-scores was only present in children of permissive parents (Figure 1d). The negative association between FF and child fruit consumption was not present in children of authoritative parents, and the negative association between EUE and child fruit consumption was not present in children of permissive parents (Figure 1e and 1f).

## Discussion

This study examined cross-sectional and longitudinal associations between children’s appetitive traits and fruit intake, snack intake, SSB intake and weight in a large, community-based sample of children in the Netherlands aged 8–11 years. It also examined whether parenting style interacted with appetite in determining child weight/intake. It replicated previous findings of positive, graded associations between food-approaching CEBQ scales and weight, and negative, graded associations between food-avoidant CEBQ scales and weight [9], [11]–[15], with the weakest associations for the EUE and FF scales.

To our knowledge, only four observational studies have related children’s appetitive behaviours to child intake, of which two used the DEBQ [22], [23] and two the CEBQ [21], [24]. These studies broadly support the hypothesized positive associations between food-approaching appetitive traits (external eating, desire to drink and enjoyment of food) with obesity-inducing behaviours (intake of SSBs and sweets) and obesity-reducing behaviours (intake of fruits and vegetables), as well as the hypothesized negative associations between food-avoidant appetitive traits (restrained eating and food neophobia) with obesity-inducing and obesity-reducing behaviours. We replicated these findings for fruit, i.e. fruit intake appeared to be positively related to food responsiveness and enjoyment of fruit, and negatively to all food-avoidant scales. However, for SSB and snack intake there were no or less consistent associations.

Because appetitive traits are known to possess a strong genetic component [32] and can be seen as stable personality traits [33], we do not expect a reverse influence of child intake/weight on appetitive behaviours. However, there is evidence that almost all parents respond to children’s appetitive traits [26] and that food responsiveness and maternal restriction are positively associated [25]. Thus, parents of food-approaching children may restrict their children on snack and SSB intake (and not on fruit intake), resulting in none or inconsistent associations between food-approaching appetite behaviours with snack and SSB intake, and positive associations with fruit intake. In addition, measurement errors may play a role in inconsistent (or lack of) findings regarding snack and SSB intake.

In general, diets rich in fruit are associated with a healthy body weight [50]–[52]; however, the food-approaching children in our study combined a higher fruit intake with a higher weight, which may indicate that these children have a greater appetite in general (also during meals) resulting in a higher total energy intake. Studies are needed in which dietary behaviours as well as total energy intake are accurately measured, to improve our understanding of e.g. the mechanisms by which appetitive traits affect weight.

Our results show that child appetitive behaviours that were associated with child intake/weight in 2009 were generally also associated with child intake/weight one year later, in 2010. However, the appetitive traits did not predict changes in child weight and hardly in child intake between 2009 and 2010; this might be explained by the follow-up period of one year, which may have been too short to express the potential gradual effect of appetitive traits on changes in child weight and intake. This explanation is supported by the finding that there was only a minimal change in average child weight and dietary behaviours between 2009 and 2010, and that almost all associations between appetitive traits and changes in child fruit, snack and SSB intake, were (although non-significant) in the same direction as the cross-sectional associations. However, another explanation is that the effect of appetitive traits on food intake/weight does not cumulate over time. Given the high tracking for weight, it is likely that food-approaching children have been growing on a higher BMI percentile and remain at that level. To establish which of these two potential explanations is most valid, requires prospective studies with a longer follow-up period. Such studies would profit from the operationalization of research models in which child dietary behaviours are modelled as mediators of the effect of appetitive traits on weight development.

We hypothesized that authoritative parenting would moderate significant associations between children’s appetite and intake/weight in a favourable way. This was supported by one finding: authoritative parenting appeared to reduce the negative effect of food fussiness on fruit consumption. We also found that neglecting parenting (characterized by low parental support and low behavioural and psychological control), strengthened the positive relation between food-approaching appetitive traits and weight. These findings underline the importance of acknowledging interaction between general parenting and child characteristics in explaining children’s food intake/weight [30]. Our results also show that inconsistencies exist regarding the optimal parenting context for child food intake/weight. Such results are also reflected in previous studies that have examined interaction between general parenting and specific parenting (e.g. restrictive feeding practices) in explaining children’s food intake and weight (see [30] for a review). The operation of moderation processes of general parenting indicates the importance of distal determinants of behaviour, that, to date, have typically been operationalized as confounders in causal chain determinants research. In contrast, we emphasize a contextual rather than causal chain orientation in examining effects of parenting on child food intake and weight.

Our findings, supported by a recent report that children are influenced by their parents’ feeding practices [25], suggest that parents are able to influence their child’s behaviour and weight, and can contribute to providing a supportive home environment for healthy child behaviour and weight. Parents of food-approaching children (i.e. children who are more vulnerable to the obesogenic environment) could help in preventing their child’s obesogenic behavioural phenotype to be expressed in high intake and weight. More insight is needed in which parental factors are essential in shaping a healthy home environment. Apart from general parenting styles and parental feeding styles, feeding practices such as availability of healthy and unhealthy foods at home, parental modelling and healthy eating parental policies should be included in future studies as potential moderators.

Although our study has the strength of combining child appetitive traits, dietary intake, weight and parenting style in one study, which is exceptional in this field of research [53], some limitations should be mentioned. First, we measured child BMI objectively, whereas dietary behaviours were measured based on Food Frequency Questionnaires, reported by parents. This may evoke social desirability bias and lead to overestimation of fruit consumption and underestimation of snacks and SSB intake [54], [55]. Had selective underreporting of snack and SSB intake indeed occurred (e.g. in food-approaching and overweight children) this would have resulted in an underestimation of the associations. Second, because our prospective study had a short follow-up period of one year and did not measure appetitive traits at both time points, the benefits of a longitudinal approach could not be fully exploited. Third, in the absence of normative data regarding parenting style dimensions, the parenting styles we constructed are relative (i.e. authoritative parents in our sample are authoritative compared to other parents in our sample). Finally, dropout analyses showed selective dropout on ethnicity; however, as this was not a main predictor and was controlled for, this probably had no effect on our results.

### Conclusion

Food-approaching appetitive traits were positively, while food-avoidant appetitive traits were negatively associated with child BMI z-scores and fruit intake. There were no (or less consistent) associations between appetitive traits and snack or SSB intake. Early assessment of appetitive traits could be used to identify food-approaching children, who are more vulnerable to the obesogenic environment and susceptible to overweight. Authoritative parenting appeared to influence fruit consumption of fussy eaters in a favourable way, while neglecting parenting appeared to influence child weight in a negative way. This makes parents a promising target group for preventive interventions aimed at influencing the effect of appetitive traits on child weight and dietary intake. However, more prospective studies with accurate measures of child appetitive traits, dietary behaviours, BMI and parenting style are needed to improve our understanding of the mechanisms by which appetitive traits affect dietary intake and weight.

## References

[1] Wang Y, Lobstein T (2006) Worldwide trends in childhood overweight and obesity. Int J Pediatr Obes 1: 11–25. 17902211 10.1080/17477160600586747

[2] Speakman JR (2004) Obesity: The Integrated Roles of Environment and Genetics. J Nutr 134: 2090S–2105S. 15284410 10.1093/jn/134.8.2090S

[3] Wardle J, Carnell S, Haworth CM, Plomin R (2008) Evidence for a strong genetic influence on childhood adiposity despite the force of the obesogenic environment. Am J Clin Nutr 87: 398–404. 18258631 10.1093/ajcn/87.2.398

[4] Carnell S, Wardle J (2008) Appetite and adiposity in children: evidence for a behavioral susceptibility theory of obesity. Am J Clin Nutr 88: 22–29. 18614720 10.1093/ajcn/88.1.22

[5] Wardle J, Guthrie C, Sanderson S, Birch L, Plomin R (2001) Food and activity preferences in children of lean and obese parents. Int J Obes Rel Metab Disord 25: 971–977. 10.1038/sj.ijo.0801661 11443494

[6] Carnell S, Wardle J (2007) Measuring behavioural susceptibility to obesity: validation of the child eating behaviour questionnaire. Appetite 48: 104–113. 16962207 10.1016/j.appet.2006.07.075

[7] Braet C, Claus L, Goossens L, Moens E, Van Vlierberghe, et al (2008) Differences in eating style between overweight and normal-weight youngsters. J Health Psychol 13: 733–743. 18697886 10.1177/1359105308093850

[8] Carnell S, Wardle J (2009) Appetitive traits in children. New evidence for associations with weight and a common, obesity-associated genetic variant. Appetite 53: 260–263. 19635515 10.1016/j.appet.2009.07.014

[9] Webber L, Hill C, Saxton J, Van Jaarsveld CHM, Wardle J (2009) Eating behaviour and weight in children. Int J Obesity 33: 21–28. 10.1038/ijo.2008.219 PMC2817450 19002146

[10] Van Strien T, Oosterveld P (2008) The children’s DEBQ for assessment of restrained, emotional, and external eating in 7- to 12-year-old children. Int J Eat Disorder 41: 72–81. 10.1002/eat.20424 17634965

[11] Viana V, Sinde S, Saxton JC (2008) Children’s Eating Behaviour Questionnaire: associations with BMI in Portuguese children. Brit J Nutr 100: 445–450. 18275626 10.1017/S0007114508894391

[12] Sleddens EF, Kremers SP, Thijs C (2008) The Children’s Eating Behaviour Questionnaire: factorial validity and association with Body Mass Index in Dutch children aged 6–7. Int J Behav Nutr Phys Act 5: 49. 18937832 10.1186/1479-5868-5-49 PMC2612017

[13] Santos JL, Ho-Urriola JA, González A, Smalley SV, Domínguez-Vásquez P, et al (2011) Association between eating behavior scores and obesity in Chilean children. Nutr J 10: 108. 21985269 10.1186/1475-2891-10-108 PMC3213088

[14] Croker H, Cooke L, Wardle J (2011) Appetitive behaviours of children attending obesity treatment. Appetite 57: 525–529. 21658420 10.1016/j.appet.2011.05.320

[15] Spence JC, Carson V, Casey L, Boule N (2011) Examining behavioural susceptibility to obesity among Canadian pre-school children: the role of eating behaviours. Int J Pediatr Obes 6: e501–e507. 20831463 10.3109/17477166.2010.512087

[16] Archer LA, Rosenbaum PL, Streiner DL (1991) The Children’s Eating Behavior Inventory: Reliability and Validity Results. J Pediatr Psychol 16: 629–642. 1744810 10.1093/jpepsy/16.5.629

[17] Wardle J, Guthrie CA, Sanderson S, Rapoport L (2001) Development of the Children’s Eating Behaviour Questionnaire. J Child Psychol Psyc 42: 963–970. 10.1111/1469-7610.00792 11693591

[18] Powers SW, Chamberlin LA, van Schaick KB, Sherman SN, Whitaker RC (2006) Maternal feeding strategies, child eating behaviors, and child BMI in low-income African-American preschoolers. Obesity 14: 2026–2033. 17135620 10.1038/oby.2006.237

[19] Wudy SA, Hagemann S, Dempfle A, Ringler G, Blum WF, et al (2005) Children with idiopathic short stature are poor eaters and have decreased body mass index. Pediatrics 116: e52–57. 15995019 10.1542/peds.2004-1684

[20] Elfhag K, Morey LC (2008) Personality traits and eating behavior in the obese: poor self-control in emotional and external eating but personality assets in restrained eating. Eating behaviors 9: 285–293. 18549987 10.1016/j.eatbeh.2007.10.003

[21] Sweetman C, Wardle J, Cooke L (2008) Soft drinks and “desire to drink” in preschoolers. Int J Behav Nutr Phys Act 5: 60. 19055714 10.1186/1479-5868-5-60 PMC2612018

[22] Elfhag K, Tholin S, Rasmussen F (2008) Consumption of fruit, vegetables, sweets and soft drinks are associated with psychological dimensions of eating behaviour in parents and their 12-year-old children. Public Health Nutr 11: 914–923. 18498675 10.1017/S1368980008002371

[23] Elfhag K, Tynelius P, Rasmussen F (2007) Sugar-sweetened and artificially sweetened soft drinks in association to restrained, external and emotional eating. Physiol behav 91: 191–195. 17434544 10.1016/j.physbeh.2007.02.005

[24] Cooke LJ, Wardle J, Gibson E, Sapochnik M, Sheiham A, et al (2004) Demographic, familial and trait predictors of fruit and vegetable consumption by pre-school children. Public Health Nutr 7: 295–302. 15003137 10.1079/PHN2003527

[25] Webber L, Cooke L, Hill C, Wardle J (2010) Associations between children’s appetitive traits and maternal feeding practices. J Am Diet Assoc 110: 1718–1722. 21034886 10.1016/j.jada.2010.08.007

[26] Carnell S, Cooke L, Cheng R, Robbins A, Wardle J (2011) Parental feeding behaviours and motivations. A qualitative study in mothers of UK pre-schoolers. Appetite 57: 665–673. 21884741 10.1016/j.appet.2011.08.009 PMC5578399

[27] Pinard CA, Yaroch AL, Hart MH, Serrano EL, McFerren MM, et al (2011) Measures of the home environment related to childhood obesity: a systematic review. Public Health Nutr 7: 1–13. 10.1017/S1368980011002059 21899786

[28] Kral TVE, Rauh EM (2010) Eating behaviors of children in the context of their family environment. Physiol behav 100: 567–573. 20457172 10.1016/j.physbeh.2010.04.031 PMC2896260

[29] Darling N, Steinberg L (1993) Parenting style as context: An integrative model. Psychol Bull 113: 487–496.

[30] Sleddens E, Gerards S, Thijs C, De Vries N, Kremers S (2011) General parenting, childhood overweight and obesity-inducing behaviors: a review. Int J Pediatr Obes 6: e12–27. 21657834 10.3109/17477166.2011.566339

[31] Bronfenbrenner U (1979) The ecology of human development: experiments by nature and design. Cambridge, MA : Harvard University Press.

[32] Wardle J, Carnell S (2009) Appetite is a heritable phenotype associated with adiposity. Ann Behav Med 38: 25–30. 10.1007/s12160-009-9116-5 19730964

[33] Ashcroft J, Semmler C, Carnell S, van Jaarsveld CHM, Wardle J (2008) Continuity and stability of eating behaviour traits in children. Eur J Clin Nutr 62: 985–990. 17684526 10.1038/sj.ejcn.1602855

[34] van Jaarsveld CHM, Llewellyn CH, Johnson L, Wardle J (2011) Prospective associations between appetitive traits and weight gain in infancy. Am J Clin Nutr 94: 1562–1567. 22071702 10.3945/ajcn.111.015818

[35] Rennie KL, Johnson L, Jebb SA (2005) Behavioural determinants of obesity. Best Pract Res Clin Endocrinol Metab 19: 343–358. 16150379 10.1016/j.beem.2005.04.003

[36] Brug J, van Stralen MM, Te Velde SJ, Chinapaw MJM, De Bourdeaudhuij I, et al (2012) Differences in weight status and energy-balance related behaviors among schoolchildren across Europe: the ENERGY-project. PloS one 7: e34742. 22558098 10.1371/journal.pone.0034742 PMC3338827

[37] Bogers RP, Van Assema P, Kester AD, Westerterp KR, Dagnelie PC (2004) Reproducibility, validity, and responsiveness to change of a short questionnaire for measuring fruit and vegetable intake. Am J Epidem 159: 900–909. 10.1093/aje/kwh123 15105183

[38] Haraldsdottir J, Thorsdottir I, de Almeida MDV, Maes L, Pérez Rodrigo C, et al (2005) Validity and reproducibility of a precoded questionnaire to assess fruit and vegetable intake in European 11- to 12-year-old schoolchildren. Ann Nutr Metab 49: 221–227. 16088085 10.1159/000087276

[39] Cole TJ, Bellizzi MC, Flegal KM, Dietz WH (2000) Establishing a standard definition for child overweight and obesity worldwide: international survey. Brit Med J 320: 1–6. 10797032 10.1136/bmj.320.7244.1240 PMC27365

[40] Fredriks AM, Van Buuren S, Wit JM, Verloove-Vanhorick SP (2000) Body index measurements in 1996–7 compared with 1980. Arc Dis Child 82: 107–112. 10.1136/adc.82.2.107 PMC1718204 10648362

[41] Beyers W, Goossens L (1999) Emotional autonomy, psychosocial adjustment and parenting: Interactions, moderating and mediating effects. J Adolescence 22: 753–769. 10.1006/jado.1999.0268 10579888

[42] Steinberg L, Elmen JD, Mounts NS (1989) Authoritative parenting, psychosocial maturity, and academic success among adolescents. Child Dev 60: 1424–1436. 2612251 10.1111/j.1467-8624.1989.tb04014.x

[43] Lamborn SD, Mounts NS, Steinberg L, Dornbusch SM (1991) Patterns of competence and adjustment among adolescents from authoritative, authoritarian, indulgent, and neglectful families. Child Dev 62: 1049–1065. 1756655 10.1111/j.1467-8624.1991.tb01588.x

[44] Huver RME, Engels RCME, Vermulst AA, De Vries H (2007) Is parenting style a context for smoking-specific parenting practices? Drug Alcohol Depen 89: 116–125. 10.1016/j.drugalcdep.2006.12.005 17300879

[45] Kremers SP, Brug J, de Vries H, Engels RCM (2003) Parenting style and adolescent fruit consumption. Appetite 41: 43–50. 12880620 10.1016/s0195-6663(03)00038-2

[46] Pearson N, Atkin AJ, Biddle SJ, Gorely T, Edwardson C (2010) Parenting styles, family structure and adolescent dietary behaviour. Public Health Nutr 13: 1245–1253. 19954574 10.1017/S1368980009992217

[47] Goossens L, Beyers W (1999) Parenting style and adolescent adjustment: a two-year longitudinal study. Paper presented at 9^th^ European Conference on Developmental Psychology, Island of Spetses, Greece.

[48] Huver RME, Engels RCME, Van Breukelen G, de Vries H (2007) Parenting style and adolescent smoking cognitions and behaviour. Psychology and Health 22: 575–593.

[49] Statistics Netherlands (2000) Standaarddefinitie allochtonen. In Hoe doet het CBS dat nou? Voorburg, The Netherlands: 24–25.

[50] Alinia S, Hels O, Tetens I (2009) The potential association between fruit intake and body weight – a review. Obes Rev 10: 639–647. 19413705 10.1111/j.1467-789X.2009.00582.x

[51] World Health Organization (2002) The World Health Report. Reducing Risks, Promoting Healthy Life. Geneva: WHO.

[52] World Health Organization (2003) Diet, Nutrition and the Prevention of Chronic Diseases. Report of a Joint WHO/FAO Expert Consultation. Geneva: WHO.

[53] Ventura AK, Birch LL (2008) Does parenting affect children’s eating and weight status? Int J Behav Nutr Phys Act 5: 15. 18346282 10.1186/1479-5868-5-15 PMC2276506

[54] Baranowski T, Smith M, Baranowski J, Wang DT, Doyle C, et al (1997) Low validity of a seven-item fruit and vegetable food frequency questionnaire among third-grade students. J Am Diet Ass 97: 66–68. 8990421 10.1016/S0002-8223(97)00022-9

[55] Van Assema P, Brug J, Ronda G, Steenhuis I, Oenema A (2002) A short Dutch questionnaire to measure fruit and vegetable intake: relative validity among adults and adolescents. Nutrition and Health 16: 85–106. 12102370 10.1177/026010600201600203

